# Machine-learning-based contrast-enhanced computed tomography radiomic analysis for categorization of ovarian tumors

**DOI:** 10.3389/fonc.2022.934735

**Published:** 2022-08-09

**Authors:** Jiaojiao Li, Tianzhu Zhang, Juanwei Ma, Ningnannan Zhang, Zhang Zhang, Zhaoxiang Ye

**Affiliations:** ^1^ Department of Radiology, National Clinical Research Center for Cancer, Tianjin Key Laboratory of Cancer Prevention and Therapy, Tianjin’s Clinical Research Center for Cancer, Tianjin Medical University Cancer Institute and Hospital, Tianjin, China; ^2^ Department of Radiology, First Affiliated Hospital of Hebei North University, Zhangjiakou, China; ^3^ Department of Radiology, Tianjin Medical University General Hospital, Tianjin, China

**Keywords:** radiomics, ovarian neoplasms, computed tomography, machine learning, classification

## Abstract

**Objectives:**

This study aims to evaluate the diagnostic performance of machine-learning-based contrast-enhanced CT radiomic analysis for categorizing benign and malignant ovarian tumors.

**Methods:**

A total of 1,329 patients with ovarian tumors were randomly divided into a training cohort (N=930) and a validation cohort (N=399). All tumors were resected, and pathological findings were confirmed. Radiomic features were extracted from the portal venous phase images of contrast-enhanced CT. The clinical predictors included age, CA-125, HE-4, ascites, and margin of tumor. Both radiomics model (including selected radiomic features) and mixed model (incorporating selected radiomic features and clinical predictors) were constructed respectively. Six classifiers [k-nearest neighbor (KNN), support vector machines (SVM), random forest (RF), logistic regression (LR), multi-layer perceptron (MLP), and eXtreme Gradient Boosting (XGBoost)] were used for each model. The mean relative standard deviation (RSD) and area under the receiver operating characteristic curve (AUC) were applied to evaluate and select the best classifiers. Then, the performances of the two models with selected classifiers were assessed in the validation cohort.

**Results:**

The MLP classifier with the least RSD (1.21 and 0.53, respectively) was selected as the best classifier in both radiomics and mixed models. The two models with MLP classifier performed well in the validation cohort, with the AUCs of 0.91 and 0.96 and with accuracies (ACCs) of 0.83 and 0.87, respectively. The Delong test showed that the AUC of mixed model was statistically different from that of radiomics model (*p*<0.001).

**Conclusions:**

Machine-learning-based CT radiomic analysis could categorize ovarian tumors with good performance preoperatively. The mixed model with MLP classifier may be a potential tool in clinical applications.

## Introduction

Ovarian cancer (OC) remains the third most common gynecological malignant tumor responsible for gynecological-cancer-related deaths (1). Patients with OC are often treated with cytoreductive surgery followed by chemotherapy or neoadjuvant chemotherapy followed by interval debulking (2). Conservative management can reduce unnecessary operative costs, decrease long-term surgical complications, and keep fertility for patients with asymptomatic benign ovarian masses (3, 4). Nevertheless, ovarian tumors were often detected incidentally, and most of them were addressed with surgery and proven to be benign (4). Therefore, characterizing the ovarian masses and assessing the possible malignant diseases would be critical for personalized treatment and follow-up plans.

Traditional imaging techniques have been widely applied for classifying ovarian tumors and monitoring treatment response in patients with OC. Specifically, ultrasound (US) is used as the first-line imaging modality for the assessment and characterization of ovarian tumors, and magnetic resonance imaging (MRI) was recommended as the problem-solving tool for the sonographically indeterminate adnexal masses (5). In addition, CT is regarded as the optimal selection for preoperative staging of OC according to the European Society of Urogenital Radiology guidelines (6); however, its role in the differentiation between benign and malignant ovarian masses is limited. In contrast to its suboptimal diagnostic performance, CT with its high spatial resolution and wide availability has been widely used in the incidental initial detection in routine clinical practice. The diagnosis of ovarian tumors mainly depended on these imaging modalities and subjective imaging assessment. However, it is limited in heterogeneity detection of ovarian masses. Therefore, it is crucial to develop a precise, objective, and non-invasive approach to preoperative categorization of ovarian tumors based on CT images.

Radiomics, allowing for high-dimensional quantitative data extraction from medical images, has shown significant power for tumor detection and classification along with objective support for clinical treatment strategy (7, 8). In brief, radiomics can offer additional diagnostic information that cannot be visible to the human eyes (9), and the information may remedy the limitation of lower soft tissue resolution in CT images. Recent studies have shown that radiomics images could distinguish benign from malignant masses with favorable performance using CT (10–13). Furthermore, machine learning, an emerging data mining approach, has offered various useful methodologies to efficiently and effectively construct accurate models for prediction based on a large number of variables (14–16). Combined with machine learning algorithms, radiomics techniques have been implemented for various cancer diagnoses (16–18).

The study aims to apply 3D radiomic analysis to preoperatively categorize benign and malignant ovarian tumors using different machine learning classifiers to build the best radiomics model and to develop and validate the mixed model by combining the radiomic and clinical features.

## Materials and methods

### Patients

**Figure 1:**
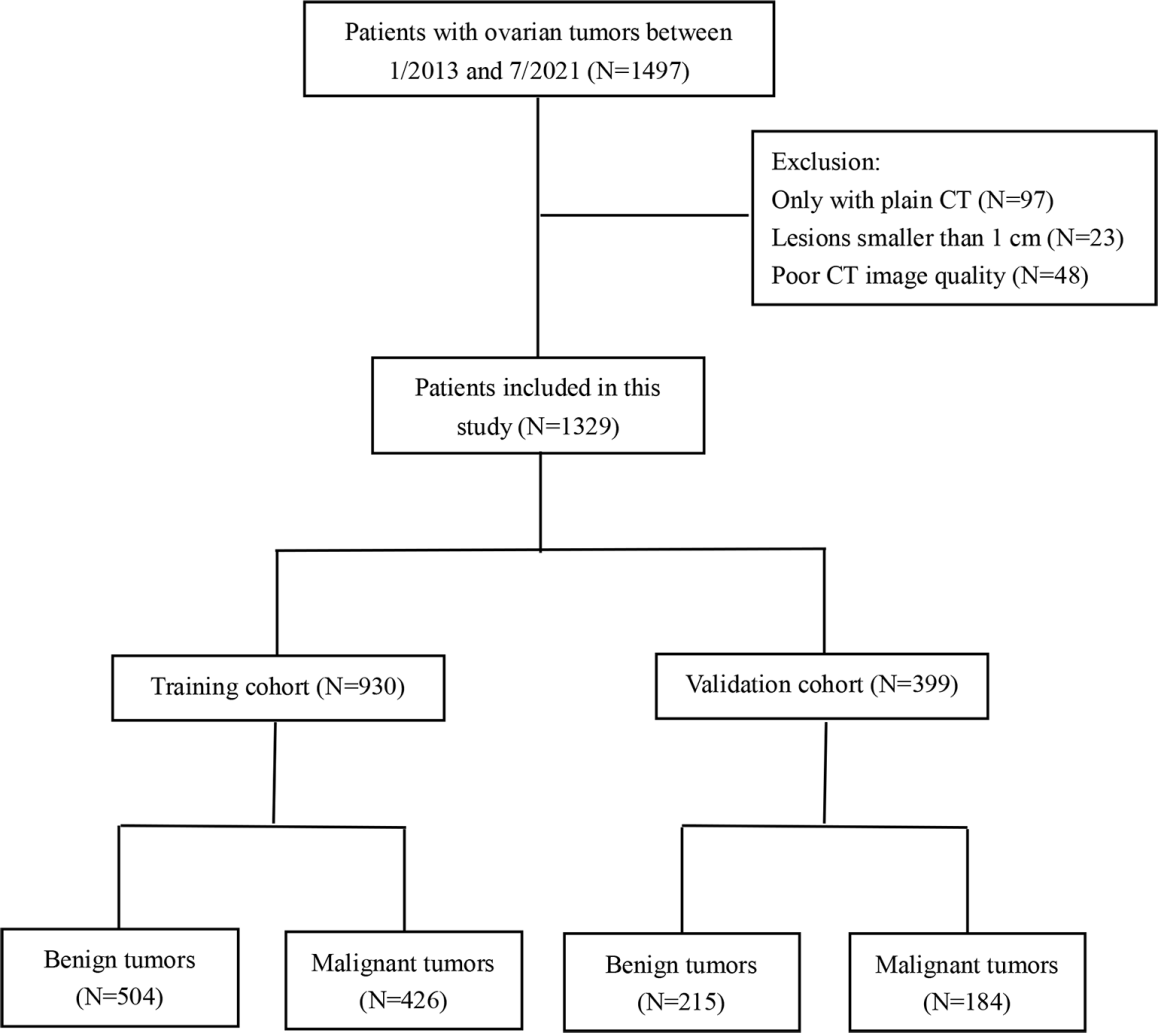
The patient selection workflow.

This retrospective study was approved by the review board of Tianjin Medical University Cancer Hospital (approval no. bc2022048), and informed consent was waived. All the clinical records have been desensitized. We included patients with ovarian tumors from January 2013 to July 2021. The inclusion criteria were as follows: (1) availability of pretreatment contrast-enhanced CT images and (2) definite histological diagnosis of ovarian tumors. The exclusion criteria were as follows: (1) patients with lesions smaller than 1 cm and (2) poor image quality or serious artifacts. Finally, 1,329 patients were selected and included in this study. In addition, all the patients were randomly divided into a training cohort (N=930) and a validation cohort (N=399) with a ratio of 7:3. A flowchart for the recruitment of patients is shown in **Figure 1**.

### Clinical data

The clinical data of all patients were retrospectively analyzed from our picture archiving and communication system (PACS), including clinical baseline and CT-reported data. CA-125 and HE-4 are widely used to diagnose and monitor OC (19). The presence of ill-defined margin of the masses and a large number of ascites is an indication of ovarian malignant tumors (20, 21). Thus, the clinical baseline data including age, CA-125 level, and HE-4 level were recorded. In addition, CT-reported data including ascites and margin of tumor were recorded and evaluated by two radiologists (with 7 years of experience, JL; with 3 years of experience, TZ). The two radiologists were blinded to histological results and clinical information. Differences were resolved by consensus.

### CT image acquisition

All patients underwent an abdominal-pelvic or pelvic contrast-enhanced CT scan before treatment. Contrast-enhanced CT scans were performed using seven different CT scanners: Discovery CT750 HD (GE medical systems), Revolution EVO (GE medical systems), Optima CT680 Expert (GE medical systems), LightSpeed 16 (GE medical systems), SOMATOM Definition AS+ (SIEMENS), SOMATOM Definition Drive (SIEMENS), and IQon Spectral CT (Philips). The acquisition parameters were as follows: matrix, 512×512; tube voltage, 120 kVp; auto tube current; and thickness, 1.0–2.5 mm. The volume of contrast medium was weight based (body weight in kg × 1.5 ml) with an upper limit of 150 ml, a concentration of 270–350 mg/ml iodine through an antecubital vein at the rate of 2.5–2.8 ml/s. The portal venous phase of contrast-enhanced CT images was retrieved at a 55–60 s delay after contrast medium injection.

### Tumor segmentation

In the case of bilateral ovarian tumors, only the larger tumor was subjected to tumor segmentation and further radiomic analysis. For each patient, the volume of interest (VOI) for ovarian tumor was delineated manually slice by slice on the portal venous phase of contrast-enhanced CT using 3D-slicer software (www.slicer.org), as illustrated in **Supplementary Figure S1**, by a radiologist (with 7 years of experience, JL). The inter- and intra-observer consistency of tumor segmentation was analyzed with 50 randomly chosen patients after 3 weeks. The same radiologist (with 7 years of experience, JL) performed a region of interest (ROI) drawing with the same method for intra-observer agreement assessment, and another radiologist (with 3 years of experience, TZ) performed ROI segmentation with the same method independently to assess inter-observer reliability.

### Extraction and selection of radiomic features

The radiomic features were extracted by PyRadiomics, and additional details are in **Supplementary Materials 1.1**. A total of 1,316 radiomic features were extracted from original and filtered images (five Laplace of Gaussian filter and eight wavelet transform). The radiomic features included 14 shape-based features, 18 first-order statistic features, 24 gray-level co-occurrence matrix (GLCM) features, 16 gray-level run-length matrix (GLRLM) features, 16 gray-level size zone matrix (GLSZM) features, 14 gray-level dependence matrix (GLDM) features, 5 neighboring gray-tone difference matrix (NGTDM) features, 465 Laplacian of Gaussian-filtered (LoG) features, and 744 wavelet features. The intra-class correlation coefficients (ICCs) were calculated to evaluate inter- and intra-observer repeatability of radiomic features extraction. The radiomic features with the values of ICCs >0.8 were reserved.

Extracted radiomic features were standardized by a z-score normalization, and additional details are in **Supplementary Materials 1.2**. We selected radiomic features based on the following steps in the training cohorts. First, the top 10% of best features calculated by univariate analysis using the SelectPercentile were selected. Then, Pearson or Spearman correlation matrices were utilized to assess the correlation between the radiomic features where a correlation coefficient >0.8 was considered redundant. Finally, a wrapper feature selection method based on RF classifier was used to choose the best predictive features.

### Construction of radiomics and mixed model

Radiomics model based on selected radiomic features was established. Univariate analysis and multivariate logistic regression analysis were conducted to select independent predictors from clinical variables. Then, wrapper feature selection based on RF classifier was further used to select the significant features from the selected radiomic features and independent clinical predictors, based on which a mixed model was built.

### Model validation and evaluation

Six supervised machine learning classifiers were used for the radiomics and mixed model: k-nearest neighbor (KNN), support vector machines (SVMs), random forest (RF), logistic regression (LR), multi-layer perceptron (MLP), and eXtreme Gradient Boosting (XGBoost). The fivefold cross-validation for each classifier was applied to the training cohort, and a StratifiedKFold iterator in scikit-learn was used. To select the best machine learning classifier, relative standard deviation (RSD) was employed to quantify the stability of the six classifiers. RSD (22) was defined as the ratio between the standard deviation and mean of the fivefold cross-validation AUC values in the training cohort:


$$\text{RSD}=\frac{sdAUC}{\text{meanAUC}}*100$$

The lower the RSD value, the higher the stability of the classifier. Similar to a previous study (23), we used the median values of area under the receiver-operating characteristic curve (AUC) and RSD as thresholds to assess and select the classifiers among six classifiers in the training cohort. The classifiers with RSD ≤1.64 and AUC ≥0.91, and with RSD ≤0.94 and AUC ≥0.94 were considered as highly reliable and accurate in the radiomics and mixed models, respectively. Moreover, the classifier with the highest AUC in the validation cohort was considered as the best classifier. In addition, confusion matrix-derived metrics, including accuracy (ACC), sensitivity (Sens), specificity (Spec), positive predictive value (PPV), and negative predictive value (NPV) of two models were calculated to further assess the two models with the best classifier. The difference in the AUC values between the two models with the best classifier was tested using the *p*-value of DeLong (D) test. The one with the higher AUC was identified as the best model.

### Human readout

All CT images from the validation cohort were evaluated by a senior (ZZ, with 12 years of working experience) in random order in the radiology department. The senior was blinded to the research design, clinical information, and background.

### Statistical analysis

In this study, differences in patients’ clinical predictors between training and validation cohorts were compared by using the Mann–Whitney *U* test for continuous variables and the chi-square test for categorical variables. The Mann–Whitney *U* test or chi-square test was utilized to identify independent clinical predictors for the univariate analysis. Binary logistic regression analysis was used for multivariate logistic regression analysis. A two-tailed *p*<0.05 was considered statistically significant. Statistical analysis was performed using MedCalc 18.2.1. The feature selection and model building were performed using Python 3.7. The boxplot of ICCs of radiomics features and heatmap were drawn by R software (version 4.1.2).

## Results

### Patients’ demographics and clinical characteristics

Histological characteristics of ovarian tumors after surgery are summarized in **Table 1**. The study consisted of 719 patients with benign ovarian tumors and 610 patients with malignant ovarian tumors. The clinical baseline and CT-reported data of patients are presented in **Table 2**. There were no significant differences in patient age, level of CA-125, level of HE-4, ascites, and the margin of tumor between the training and validation cohorts (all *p*>0.05).

**Table 1:** Histopathological characteristics of ovarian tumors included in the study.

Variable	Patients of ovarian tumors (*N*=1329)
Benign	719 (54.10%)
Malignant	610 (45.90%)
Histological type of benign tumors:	
Serous cystoadenoma/cystoadenofibroma	116 (16.13%)
Mucinous cystoadenoma	110 (15.30%)
Teratoma	198 (27.54%)
Fibroma/fibrothecoma	105 (14.60%)
Endometriotic cyst/endometrioid tumor	144 (20.03%)
Seromucinous cystadenoma	13 (1.81%)
Brenner tumor	1 (0.14%)
Other benign tumors	32 (4.45%)
Histological type of malignant tumors:	
High grade serous ovarian cancer	305 (50.00%)
Low grade serous ovarian cancer	23 (3.77%)
Borderline tumor	78 (12.79%)
Mucinous carcinoma	18 (2.95%)
Endometrioid ovarian cancer	60 (9.84%)
Clear cell ovarian cancer	61 (10.00%)
Carcinosarcoma	2 (0.33%)
Undifferentiated carcinoma	1 (0.16%)
Ovarian metastases from other tumors	25 (4.10%)
Granulosa cell ovarian tumor	20 (3.28%)
Immature teratoma	11 (1.80%)
Other malignant tumors	6 (0.98%)

Results are presented as N (%).

**Table 2:** Patients characteristics.

Characteristics	Training cohort (*N*=930)	Validation cohort (*N*=399)	*p*-value
Benign tumors, N (%)	504 (54.19%)	215 (53.88%)	–
Malignant tumors, N (%)	426 (45.81%)	184 (46.12%)	–
Age, median (IQR)	51.00 (40.00,59.00)	50.00 (41.00,59.00)	0.70^1^
HE-4, median (IQR)	58.90 (46.95,133.00)	58.44 (45.39,138.78)	0.76^1^
CA-125, median (IQR)	51.48 (16.00,253.00)	52.30 (17.53,258.05)	0.56^1^
CT-reported margin, N (%)			
Well defined	624 (67.10%)	261 (65.41%)	0.55^2^
Ill defined	306 (32.90%)	138 (35.59%)	
CT-reported ascites, N (%)			
Absent	371 (39.89%)	165 (41.35%)	0.62^2^
Present	559 (60.11%)	234 (58.65%)	

Results are presented as N (%).

IQR, interquartile range.

^1^Mann–Whitney U test.

^2^Chi-square test.

The results of the univariate analysis and multivariate logistic regression analysis are shown in **Table 3**. Patients with benign ovarian tumors were younger than those with malignant ovarian tumors (*p*<0.0001). The HE-4 and CA-125 levels in patients with malignant ovarian tumors were higher than those in patients with benign ovarian tumors (both *p*<0.0001). The women with benign ovarian tumors had fewer ascites (*p*<0.0001), and the margin of tumor was better defined in those (*p*<0.0001). Multivariate regression logistic analysis showed no significant differences in age and level of CA-125 (both *p*>0.05). Finally, the level of HE-4, ascites, and margin were selected as independent clinical predictors related to benign and malignant ovarian tumors.

**Table 3:** Results of univariate analysis and multivariate logistic regression analysis for clinical predictors in the training cohort.

Clinical predictors	Univariate analysis			Multivariate regression analysis	
	Benign tumors	Malignant tumors	*p-*value	Odd ratio (95% CI)	*p-*value
Age (IQR)	47.00 (34.00–59.00)	54.00 (47.00–60.00)	<0.0001^1^	1.00 (0.99–1.02)	0.8328
HE-4 (IQR)	49.55 (42.80–57.53)	150.55 (69.96–372.00)	<0.0001^1^	1.04 (1.03–1.05)	<0.0001
CA-125 (IQR)	19.98 (11.86–51.09)	239.50 (68.50–715.00)	<0.0001^1^	1.00 (1.00–1.00)	0.4608
Margin, N (%)			<0.0001^2^	6.22 (3.92–9.86)	<0.0001
Well defined	461 (91.47%)	163 (38.26%)			
Ill defined	43 (8.53%)	263 (61.74%)			
Ascites, N (%)			<0.0001^2^	1.73 (1.15–2.59)	<0.0001
Absent	276 (54.76%)	95 (22.30%)			
Present	228 (45.24%)	331 (77.70%)			

Results are presented as N (%).

IQR interquartile range.

CI confidence interval.

^1^Mann–Whitney U test.

^2^chi-square test.

### Radiomic features analysis and feature selection

**Figure 2:**
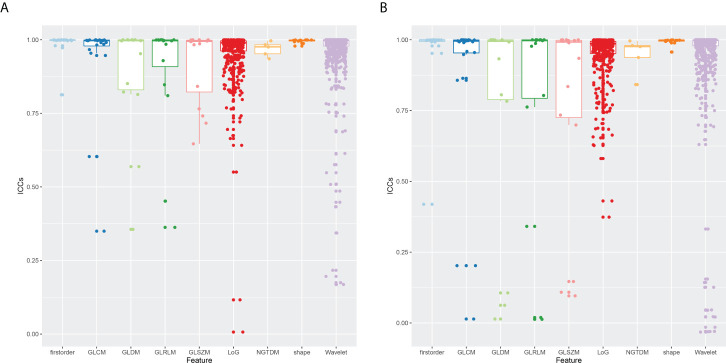
Boxplot of ICCs of radiomic features extracted from nine feature groups. **(A)** Intra-observer ICCs. **(B)** Inter-observer ICCs. ICCs, intra-class correlation coefficients.

The ICCs were calculated to assess the robustness and repeatability of radiomic features (**Figure 2**). The shape-based features, first-order statistic features, GLCM features, GLRM features, NGTDM features, LoG features, and wavelet features held high robustness and repeatability not only in the inter-observer measurement but also in the intra-observer measurement. The mean ICC values of these features were all >0.8. However, the GLSZM features (0.916 ± 0.127 *vs*. 0.783 ± 0.345) and GLDM features (0.883 ± 0.196 *vs*. 0.764 ± 0.388) had high robustness in intra-observer measurement, whereas there was less repeatability in inter-observer measurement.

Totally, 1,229 radiomic features with ICCs>0.8 including 14 shape-based features, 17 first-order statistic features, 21 features of GLCM, 12 features of GLRLM, 11 features of GLSZM, 10 features of GLDM, 5 features of NGTDM, 429 LoG features, and 710 wavelet features were retained and used for further feature selection. Then, after feature selection including univariate analysis and rapper feature selection method, nine radiomic features including one shape feature, four LoG features, and four wavelet features associated with differentiation in ovarian tumors were identified and further applied to establish models. Detailed information about feature selection and these selected radiomic features can be found in **Supplementary Material 1.3 and Supplementary Table S1**.

### Model construction and comparison

**Figure 3:**
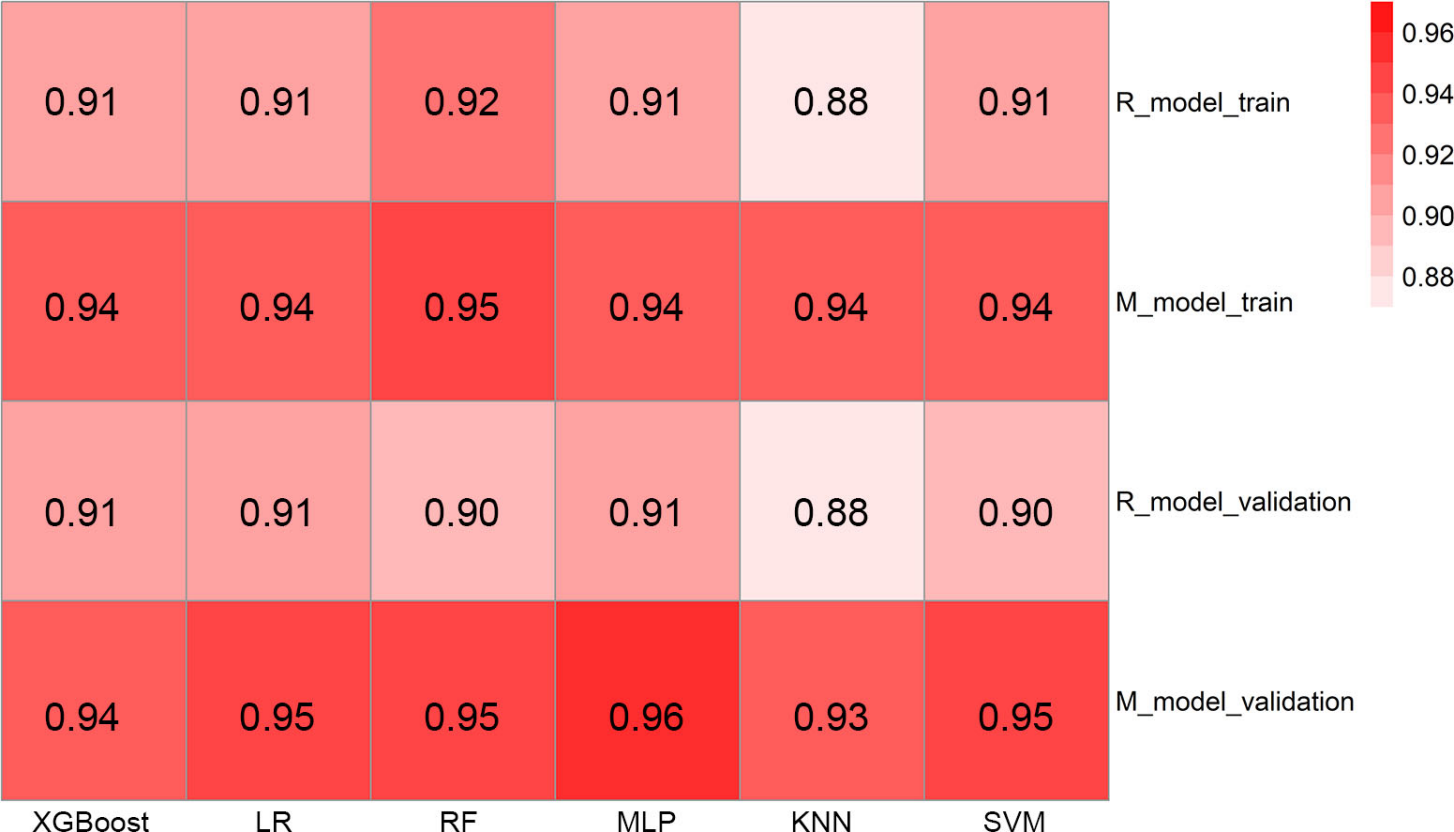
The heatmap illustrating the predictive performance (AUC) for the six classifiers. AUC, area under the receiver-operating characteristic curve; R_model_train, radiomics model in the training cohort; M_model_train, mixed model in the training cohort; R_model_validation, radiomics model in the validation cohort; M_model_validation, mixed model in the validation cohort; XGBoost, eXtreme Gradient Boosting; LR, logistic regression; RF, random forest; MLP, multi-layer perceptron; KNN, k-nearest neighbor; SVM, support vector machines.

**Figure 4:**
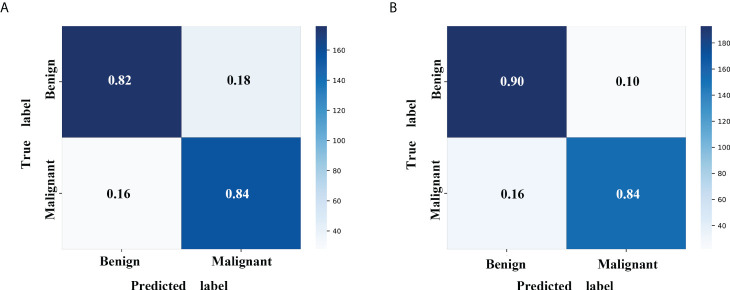
Confusion matrix with MLP classifier in the validation cohort. **(A)** The radiomics model with MLP classifier. **(B)** The mixed model with MLP classifier. MLP, multi-layer perceptron.

**Figure 5:**
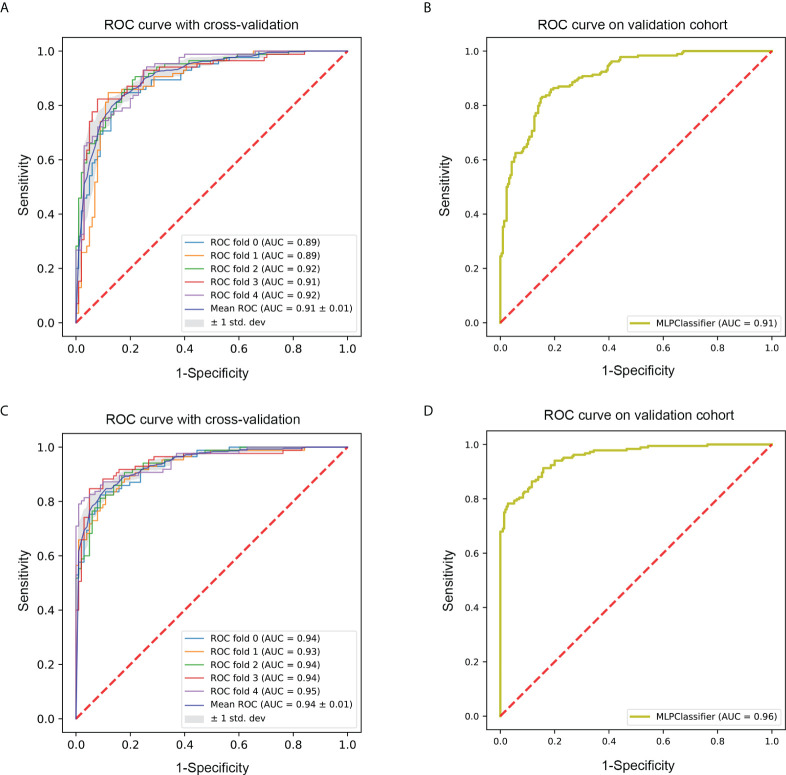
ROC curve of the optimal classifier (MLP). **(A)** The fivefold cross-validation ROC curve of radiomics model with MLP classifier in the training cohort. **(B)** The ROC curve of radiomics model with MLP classifier in the validation cohort. **(C)** The fivefold cross-validation ROC curve of mixed model with MLP classifier in the training cohort. **(D)** The ROC curve of mixed model with MLP classifier in the validation cohort. ROC, receiver-operating characteristic; MLP, multi-layer perceptron.

A radiomics model based on the nine selected radiomic features was developed. HE-4 level, margin, and six radiomic features were determined by the wrapper method based on RF classifier and selected as independent predictors to construct the mixed model. These predictors have been detailed in **Supplementary Material 1.4 and Supplementary Table S2**. The six classifiers including KNN, SVM, RF, LG, MLP, and XGBoost were evaluated in the radiomics and mixed model. The RSDs of the six classifiers in the radiomics and mixed model are shown in **Table 4**. The heatmap of the mean AUC values for the six classifiers in the radiomics and mixed model is presented in **Figure 3**. According to the criteria for RSD ≤1.64 and AUC ≥0.91, and RSD ≤0.94 and AUC ≥0.94 in the radiomics and mixed models, respectively, the classifiers (XGBoost, MLP, and SVM) in the radiomics model and the classifiers (LR, MLP, and SVM) in the mixed model were selected. The selected classifiers were then applied to the validation cohort. Among these selected classifiers, the MLP had the highest AUC in both the radiomics and mixed models, which was chosen as the best classifier. Therefore, MLP was chosen as the machine learning classifier for constructing the radiomics and mixed models. The performances (AUC, ACC, Sens, Spec, PPV, and NPV) of radiomics and mixed model with the six classifiers are listed in **Table 5** and **Figure 3**. **Figure 4** illustrates the confusion matrix of the optimal classifier of MLP in the radiomics and mixed model. The ACCs were 0.83 and 0.87 for the radiomics and mixed model with the optimal classifier. The Sens, Spec, PPV, and NPV of MLP classifier were 0.84, 0.82, 0.86, and 0.80 in the radiomics model, and 0.84, 0.84, 0.87, and 0.88 in the mixed model, respectively. Moreover, we compared the diagnostic performance of the aforementioned radiomics and mixed models with that derived from the visual inspection of senior radiologist. As shown in **Table 5**, the AUC was 0.78 for the senior radiologist, and it was inferior to the AUCs of radiomics and mixed models. DeLong test showed statistically significant differences between senior radiologist’s evaluation and radiomics model (D=5.87, *p*<0.001) and mixed model (D=9.03, *p*<0.001) separately. The illustration of the fivefold cross-validation receiver-operating characteristic (ROC) curve for the training cohort and ROC curve for the validation cohort in the radiomics and mixed model with the optimal classifier is presented in **Figure 5**. The values of the AUC of the two models with the optimal classifier on the validation cohort were compared by the Delong test, and the result showed a significant difference (D=−4.36, *p*<0.001). The mixed model with MLP classifier was the best.

**Table 4:** The RSD of classifiers for radiomics and mixed model in the training cohort.

	RSD					
	XGBoost	LR	RF	MLP	KNN	SVM
Radiomics model	1.64	1.98	1.75	1.21	1.92	1.32
Mixed model	1.51	0.75	0.95	0.53	1.28	0.64

RSD, relative standard deviation; XGBoost, eXtreme Gradient Boosting; LR, logistic regression; RF, random forest; MLP, multi-layer perceptron; KNN, k-nearest neighbor; SVM, support vector machines.

**Table 5:** Diagnostic performances of the classifiers for radiomics and mixed model and the senior radiologist in the validation cohort.

Classifiers/Models	AUC	ACC	Sens	Spec	PPV	NPV
XGBoost						
Radiomics model Mixed model	0.91 0.94	0.82 0.87	0.79 0.85	0.84 0.88	0.83 0.88	0.81 0.86
LR						
Radiomics model Mixed model	0.91 0.95	0.82 0.86	0.84 0.79	0.81 0.92	0.85 0.84	0.79 0.73
RF						
Radiomics model Mixed model	0.90 0.95	0.82 0.88	0.83 0.84	0.81 0.93	0.85 0.87	0.79 0.91
MLP						
Radiomics model Mixed model	0.91 0.96	0.83 0.87	0.84 0.84	0.82 0.84	0.86 0.87	0.80 0.88
KNN						
Radiomics model Mixed model	0.88 0.93	0.82 0.85	0.73 0.81	0.90 0.89	0.80 0.85	0.86 0.86
SVM						
Radiomics model Mixed model	0.90 0.95	0.82 0.87	0.84 0.83	0.80 0.90	0.85 0.86	0.68 0.87
Senior radiologist	0.78	0.78	0.75	0.81	0.77	0.79

AUC, area under the receiver-operating characteristic curve; ACC, accuracy; Sens: sensitivity; Spec, specificity; PPV, positive predictive value; NPV, negative predictive value; XGBoost, eXtreme Gradient Boosting; LR, logistic regression; RF, random forest; MLP, multi-layer perceptron; KNN, k-nearest neighbor; SVM, support vector machines.

## Discussion

In this study, we developed and compared CT 3D radiomic analysis to categorize benign and malignant ovarian tumors preoperatively using six machine learning classifiers, and the MLP classifier exhibited the best performance. Our results showed that both radiomics and mixed models with MLP classifiers can be used for OC differentiation. Additionally, the mixed model with MLP classifier demonstrated significantly improved discrimination performance for ovarian tumors.

Traditionally, the differentiation of benign and malignant ovarian tumors was mainly dependent on the subjective analysis of US and/or MRI imaging (24). Although CT may play a useful role in differentiating ovarian masses, its performance based on subjective analysis is often imperfect. In contrast, the mixed model with MLP classifier showed superior diagnostic performance in the validation cohort (AUC=0.96) in this study. Encouragingly, the mixed model with MLP classifier could afford good classification in patients with borderline tumors regarded as low potential malignancies and further demonstrated its superiority with high PPV (0.87) and NPV (0.88) in the validation cohort. The radiomics model with MLP classifier also showed relatively high diagnostic performance in the validation cohort (AUC=0.91). Therefore, the combination of machine learning classifier and radiomic features maybe the main reason for better outperformed diagnostic performance using CT images for differentiation of ovarian tumors, which may make up for the deficiency of CT clinical application to a certain extent.

Machine learning classifiers have been extensively applied in the field of radiomic analysis and further enhanced diagnostic performance (15, 23, 25, 26). Different classifiers in machine learning have different algorithms and diagnostic efficiencies. To data, a few studies have explored radiomic analysis based on machine learning classifiers for distinguishing ovarian tumors; however, the diagnostic efficiencies of different machine learning classifiers were not compared (27, 28). In our study, we explored and compared the diagnostic values of six classifiers, namely, RF, KNN, LG, SVM, MLP, and XGBoost. KNN is a relatively simple classifier that directly calculates images to images distances. XGBoost and RF are constructed by a multitude of decision trees, and their advantages are the ease of use and ability to calculate the importance of features (29). LR is currently the most widely used machine learning classifier because of its simplicity. However, it is necessary to pay attention to its inherent limitation, such as the independence assumption to features. SVM iteratively forms a hyperplane or a set of hyperplanes in high-dimensional feature space that separates the clinical problems (30). As deep-learning is now widely used for the diagnosis of various diseases, the deep-learning or convolutional neural network (CNN) method would assist in the construction of favorable classification models. The MLP is the simplest form of an artificial neural network; it simulates the nervous system properties and biological learning functions through an adaptive process (31, 32). The present study implemented the MLP classifier and conducted the preliminary study on CNN-based classification models. Although the diagnostic value of RF was slightly higher than that of the MLP, the latter was found to be more stable than the former in the training cohort. The stability of the machine learning classifier is also very important for its model construction and clinical application (22). Therefore, the MLP was chosen to develop the radiomics and mixed model. Moreover, the two models with the MLP classifier showed the highest diagnostic efficiency than the other conventional classifiers in the validation cohort, which suggested that MLP was the preferred classifier when differentiating benign and malignant ovarian tumors.

A prior study constructed a model to classify ovarian tumors using radiomic analysis based on plain CT (33). The radiomics nomogram demonstrated the best performance in both the training (AUC=0.95) and test (AUC=0.96) cohort. External validation also showed high diagnostic performance (AUC=0.95) in the previous study. In our study, a large number of patients were used for radiomic analysis based on CT images, and borderline tumors were included. Pan et al. demonstrated that the nomogram model, combining CT radiomic and semantic features, showed the best diagnostic value to differentiate serous and mucinous ovarian cystadenomas than the radiomics-based and semantics-based models (34). The CT semantic feature of the margin, as an independent predictor, was included in the mixed model in our study. Consistent with the previous study, the mixed model possessed a higher diagnostic value than the radiomics model in which only radiomic features were involved in our study. This result further validated complementarity in the radiomic and CT semantic features. Moreover, the margin of ovarian masses was divided into two categories, namely, well-defined margin and ill-defined margin in this study, which was easily evaluated by clinicians even for inexperienced clinicians in this study. This indirectly mirrored the operability and feasibility of the mixed model in our study. Deep learning, especially CNN, has been attracting attention within radiology. For instance, Wang et al. applied CNN on routine MRI to assess the nature of ovarian tumors and found that CNN could assist radiologists in improving their diagnostic performance (35). However, manual segmentation was used for ovarian lesions segmentation in the study. Nowadays, automatic segmentation based on deep learning has been successfully implemented; nevertheless, it is still challenging for ovarian lesion on CT or MRI because the bladder or uterus are frequently confused for the ovary or ovarian lesion. Furthermore, because of the inherent limitation of the sample size for medical images, training a CNN model from scratch for one specific clinical question often does not yield satisfactory results (36). In addition, the current study also showed that HE-4 level was an independent predictor for distinguishing benign and malignant ovarian tumors. Unlike previous studies (33, 37), the level of CA-125 and HE4 were evaluated simultaneously in our study. This study showed that only the HE-4 level could be used as an independent predictor. This may be because the level of CA-125 is slightly elevated in some patients with benign ovarian tumors (38), which decreases its specificity.

The previous studies used 2D radiomic features to distinguish ovarian tumors (28, 33). 3D segmentation method was utilized to segment lesions in this study. 3D segmentation is better in capturing full information about the whole tumor and more realistically to reflect the high heterogeneity of ovarian tumors. Liu et al. have shown that 3D segmentation has better performance than 2D segmentation in distinguishing ovarian borderline tumors and epithelial cancers (39). Several previous studies have also demonstrated that radiomic features based on 3D segmentation are preferably repeatable (40) and more insensitive to manual segmentation variability (41). Furthermore, our study chose the portal venous phase CT images to extract radiomic features and demonstrated that they had favorable predictive performance. An et al. have determined the histological subtype classification in epithelial ovarian carcinoma using CT texture features extracted from the portal venous phase of contrast-enhanced CT images (42). Yu et al. compared the diagnostic performance of radiomic features extracted from different phases for discriminating between serous borderline malignant ovarian tumors, and the result indicated that the portal venous phase model showed a good predictive performance with a relatively higher AUC (43). These findings and our results may provide an idea for conducting extraction of radiomic features in future studies. In addition, this study presented an excellent diagnostic performance for categorizing ovarian tumors on seven different CT scanners, which indicated that such a heterogeneous dataset may compensate for the limitation of no external validation.

This study has some limitations. First, it was a retrospective study in a single center, which may cause a selection bias. External multi-center validation in a larger cohort is needed to verify the generalization of our model in the future. Second, our study was limited in sample size for some subgroups of ovarian tumors because of the rarity of diseases. In the future study, we will increase the sample size to validate our predictive model. Third, different feature selection methods were not compared. Finally, the VOIs were drawn manually, and the influence of subjective factors cannot be avoided completely. In the future study, semi-automatic segmentation or automatic segmentation will be used.

## Conclusion

In conclusion, this study indicated that machine learning based on CT radiomic analysis could be applied to classify benign and malignant ovarian tumors. The mixed model with MLP classifier held the best diagnostic performance, which may be used as a convenient and accurate tool in clinical settings to identify and distinguish benign and malignant ovarian tumors.

## Data availability statement

The original contributions presented in the study are included in the article/**Supplementary material**. Further inquiries can be directed to the corresponding authors.

## Ethics statement

The studies involving human participants were reviewed and approved by Tianjin Medical University Cancer Institute and Hospital. Written informed consent from the participants’ legal guardian/next of kin was not required to participate in this study in accordance with the national legislation and the institutional requirements.

## Author contributions

All the authors were involved in this study and approved the final manuscript. The individual contributions are listed below: JL: participated in data collection and analysis and drafted the manuscript. TZ: participated in data analysis and model building. JM: collected the data and performed statistical analysis. NZ: performed statistical analysis and model building. ZZ and ZY: designed the research and edited the manuscript. All authors contributed to the article and approved the submitted version.

## Funding

This work was supported by the Chinese National Key Research and Development Project (Grant Nos. 2021YFC2500400 and 2021YFC2500402), the National Natural Science Foundation of China (Grant Nos. 82071907, 81301217, and 81301202), Tianjin Health Science and Technology Project (Grant No. MS20022), Natural Science Foundation of Tianjin (Grant Nos. 18JCYBJC25100 and 18JCQNJC80200), and Wu Jieping Medical Foundation—Special Fund for Clinical Research (320.6750.2022-3-5).

## Conflict of interest

The authors declare that the research was conducted in the absence of any commercial or financial relationships that could be construed as a potential conflict of interest.

## Publisher’s note

All claims expressed in this article are solely those of the authors and do not necessarily represent those of their affiliated organizations, or those of the publisher, the editors and the reviewers. Any product that may be evaluated in this article, or claim that may be made by its manufacturer, is not guaranteed or endorsed by the publisher.
