# Supplementary material for: Machine-learning-based contrast-enhanced computed tomography radiomic analysis for categorization of ovarian tumors
Source: Front Oncol. 2022 Aug 9;12:934735. doi: 10.3389/fonc.2022.934735 (PMC9395674; doi:10.3389/fonc.2022.934735)
Supplement: Supplementary file 1 [file DataSheet_1.docx]

**1.1 Radiomic features extraction**

An open-source python package PyRadiomics was used for 3D radiomic feature extraction. The resampled voxel sizes were set to 1×1×1 mm³ voxels in order to standardize the slice thickness. Voxel array shift was set on 1000 and image intensities were binned by 25 HU. Radiomic features were extracted from the original images of tumors and images transformed by wavelet and Laplacian of Gaussian (LoG) filtration. Wavelet filtration filtered original image directionally with x, y and z directions respectively (H: High pass filter, L: Low pass filter), yielding 8 different combinations of decompositions. LoG filtration images were generated with sigma=1, 2, 3, 4 and 5 mm. The extracted radiomic features with/without filtration can be divided into 5 group: (1) shape-based features, (2) first-order statistic features, (3) second-order features, (4) LoG features, (5) wavelet features.

**1.2 Standardization of radiomic features**

Extracted radiomic features were standardized with z-score normalization before feature selection in order to allow the radiomic features with different units to be compared and weighted. The z-score normalization transform radiomic features into new scores with a standard normal distribution, where mean value of radiomic features is 0, and standard deviation (SD) is 1. The formula of z-score normalization is as follows:

$$z=\frac{x-m}{s}$$

m is the mean values of radiomics features, and s is SD.

**1.3 Details of radiomic features selection**

Totally, 1316 radiomic features were extracted from each the volume of interest (VOI) of ovarian tumor for each patient, and 1229 with higher robustness and repeatability were retained. Firstly, univariate analysis using the SelectPercentile (SP) was performed and the top 10% best predictive features were retained. In this study, analysis of SP selected 123 radiomic features. Second, feature selection of the selected 123 features was performed using pearson or spearman correlation. For the radiomic features with normal distribution, pearson correlation was used, otherwise spearman correlation was used. And a correlation coefficient greater than 0.8 was considered redundant. 23 radiomic features were selected by correlation analysis. Finally, a wrapper feature selection method based on RF classifier was used to choose the best predictive features. Using the wrapper method, radiomic features evaluation was conducted via a black box induction algorithm, and RF algorithm was selected in our study. Each time, one feature with the least importance score was removed from the feature set followed by successive iterations, and the classification accuracy was calculated. A set of radiomic features with the least number of variables and higher classification accuracy were considered as the optimal feature subset. In this study, 9 radiomic features was selected by the wrapper method. Details of the 9 radiomic features were listed in Table S1.

Table S1. Radiomic features selected for the radiomics model

| Index | Filter^a^ | Feature class | Feature |
| --- | --- | --- | --- |
| 1 | Original^b^ | Shape-based^c^ | Sphericity |
| 2 | Wavelet^d^ (LLH) | First order^e^ | Root Mean Squared |
| 3 | Wavelet (LHL) | First order | Median |
| 4 | Wavelet (LLL) | First order | Root Mean Squared |
| 5 | Wavelet (LLL) | GLDM | Dependence Entropy |
| 6 | LoG^f^ (1.0 mm) | GLDM | Large Dependence High Gray Level Emphasis |
| 7 | LoG (2.0 mm) | GLSZM | Zone Entropy |
| 8 | LoG (5.0 mm) | GLRLM | Short Run Emphasis |
| 9 | LoG (5.0 mm) | First order | 90 Percentile |

GLDM, Gray Level Dependence Matrix; GLSZM, Gray Level Size Zone Matrix Features; GLRLM, Gray Level Run Length Matrix Features; LoG, Laplacian of Gaussian-filtered.

^a^LLH, LHL and LLL represent high pass filter and low pass filter on the X, Y, Z three dimensions (H, high pass filter; L, low pass filter);

^b^Original, original images without any filter applied;

^c^Shape-based, Shaped –based features;

^d^Wavelet, wavelet filtrated image;

^e^First order, first order statistics features;

^f^LoG, Laplacian of Gaussian-filtered image.

**1.4 Details of clinical and radiomic features selection for mixed model**

The independent clinical predictors including HE-4 level, ascites and margin, and the above 9 selected radiomic features were further selected using wrapper method based on RF classifier to construct the mixed model. After selection, 2 clinical and 6 radiomic features were determined to construct the mixed model. Details of these features were listed in Table S2.

Table S2. Significant features selected for the mixed model

| Index | Filter^a^ | Feature class | Feature |
| --- | --- | --- | --- |
| 1 | Wavelet^b^ (LLH) | First order^c^ | Root Mean Squared |
| 2 | Wavelet (LLL) | First order | Root Mean Squared |
| 3 | Wavelet (LLL) | GLDM | Dependence Entropy |
| 4 | LoG^d^ (1.0 mm) | GLDM | Large Dependence High Gray Level Emphasis |
| 5 | LoG (2.0 mm) | GLSZM | Zone Entropy |
| 6 | LoG (5.0 mm) | First order | 90 Percentile |
| 7 |  |  | HE-4 level |
| 8 |  |  | Margin |

GLDM, Gray Level Dependence Matrix; GLSZM, Gray Level Size Zone Matrix Features; LoG, Laplacian of Gaussian-filtered.

^a^LLH, and LLL represent high pass filter and low pass filter on the X, Y, Z three dimensions (H, high pass filter; L, low pass filter);

^b^ Wavelet, wavelet filtrated image;

^c^ First order, first order statistics features;

^d^ LoG, Laplacian of Gaussian-filtered image.

**Supplementary Figures**

**
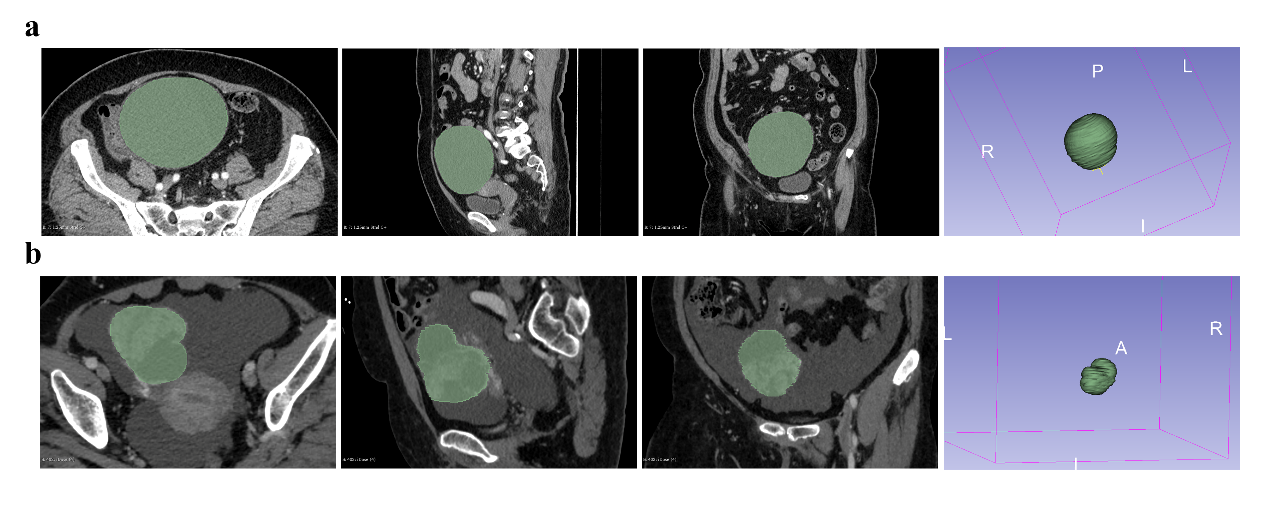
**

**Fig. S1 Examples of manual 3D segmentation of benign and malignant ovarian tumors. (A) a patient with a benign ovarian tumor. (B) a patient with a malignant ovarian tumor.**
